# Dust mite ingestion-associated, exercise-induced anaphylaxis: a case report and literature review

**DOI:** 10.1186/s13223-019-0399-1

**Published:** 2020-01-06

**Authors:** Mongkhon Sompornrattanaphan, Yanisa Jitvanitchakul, Nat Malainual, Chamard Wongsa, Aree Jameekornrak, Orathai Theankeaw, Torpong Thongngarm

**Affiliations:** 1 Division of Allergy and Clinical Immunology, Department of Medicine, Faculty of Medicine Siriraj Hospital, Mahidol University, Bangkok, Thailand; 2 Division of Hematology, Department of Medicine, Faculty of Medicine Siriraj Hospital, Mahidol University, Bangkok, Thailand; 3 Department of Parasitology, Faculty of Medicine Siriraj Hospital, Mahidol University, Bangkok, Thailand

**Keywords:** Pancake syndrome, Oral mite anaphylaxis, Food allergy, House dust mite, Exercise-induced anaphylaxis

## Abstract

**Background:**

Oral mite anaphylaxis (OMA) is a condition characterized by severe allergic reactions after ingesting food containing dust mite-contaminated flour. Physical exertion is recognized as a common trigger factor inducing anaphylaxis. The association of OMA with exercise-induced anaphylaxis has rarely been reported.

**Case presentation:**

We report a 29-year-old Thai woman who had dust mite ingestion-associated, exercise-induced anaphylaxis who tolerated the same bag of contaminated flour without exercise. A sample of contaminated cooking flour was examined under a light microscope. Living mites, *Dermatophagoides farinae*, were detected by a medical entomologist based on the morphology. We performed skin test to both mite-contaminated and newly opened Gogi® cooking flour, common aeroallergens, food allergens, and all other ingredients in the fried coconut rice cake 5 weeks after the anaphylactic episode. Specific IgE tests, using ImmunoCAP were also performed.

**Conclusions:**

Dust mite ingestion-associated, exercise-induced anaphylaxis may be misdiagnosed as wheat-dependent exercise-induced anaphylaxis and should be suspected in patients with anaphylaxis linked to food intake and exercise, but who have no apparent evidence to the index food ingredients on allergy workup.

## Background

Oral mite anaphylaxis (OMA) is a condition characterized by severe allergic reactions after ingesting food containing dust mite-contaminated flour [1, 2]. Physical exertion is recognized as a common trigger factor inducing anaphylaxis [3, 4]. The association of OMA with exercise-induced anaphylaxis has rarely been reported [5, 6]. It may be misdiagnosed as wheat-dependent exercise-induced anaphylaxis.

## Case presentation

**Fig. 1:**
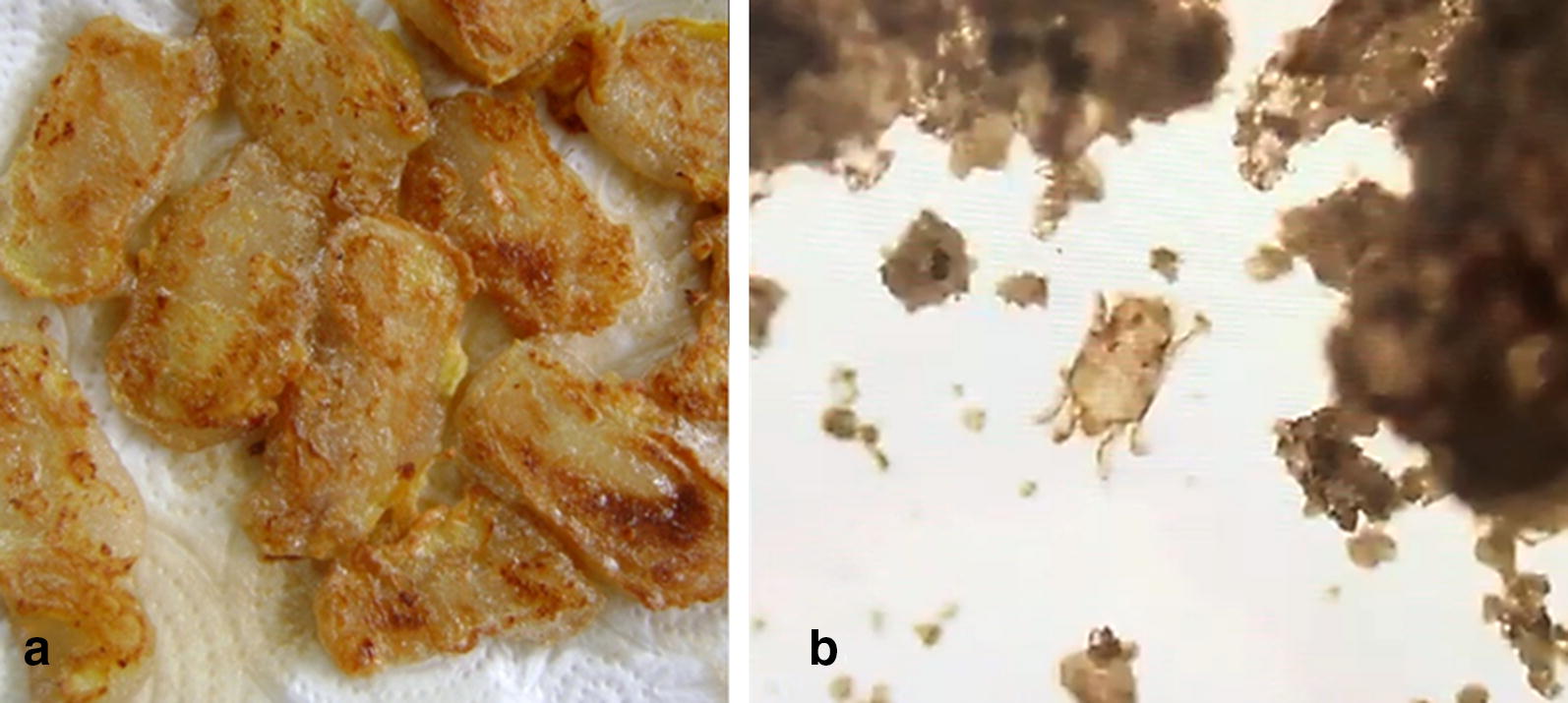
**a** Fried coconut rice cake. **b** *Dermatophagoides farinae* demonstrated by light microscopy in culprit cooking flour

We report a 29-year-old Thai woman who had dust mite ingestion-associated, exercise-induced anaphylaxis who tolerated the same bag of contaminated flour without exercise. She had moderate to severe, persistent allergic rhinitis since the age of 5, which was controlled by intranasal corticosteroid. Before the anaphylactic event occurred, she ate 10 pieces of fried coconut rice cake using mixed cooking flour (Gogi®) (Fig. 1a). Sixty minutes later, she began to jog along the road as a daily routine. Twenty minutes after jogging, she developed itchy palms and feet, followed by bilateral nasal congestion. She immediately sought medical attention for these symptoms. She then developed swollen eyelids, eye redness, watery rhinorrhea, chest tightness, and difficulty breathing.

In the emergency room, her vital signs were a blood pressure of 94/62 mmHg, a heart rate of 110 beats per minute, a respiratory rate of 24 times per minute, and an oxygen saturation of 97% on room air. Physical examination revealed angioedema of eyelids as well as generalized wheals and flares. The lungs were clear to auscultation. She was diagnosed with anaphylaxis, and food was suspected as a causative agent. Intramuscular epinephrine was administered. All symptoms improved on the first day. She had a biphasic reaction with mild recurrent eyelid angioedema the next day, which completely recovered within 24 h.

She was generally well the day before the anaphylactic event. She denied taking medications and denied history of drug allergy. She could take ibuprofen and diclofenac without any adverse reaction. One week before the anaphylactic episode, she could tolerate 15 pieces of fried coconut rice cake using the same bag of mixed cooking flour which had been opened and stored at room temperature for 2 months.

A sample of contaminated cooking flour was examined under a light microscope. Living mites, *Dermatophagoides farinae*, were detected by a medical entomologist based on the morphology (Fig. 1b). We performed skin test to both mite-contaminated and newly opened Gogi® cooking flour, common aeroallergens, food allergens, and all other ingredients in the fried coconut rice cake 5 weeks after the anaphylactic episode. Specific IgE tests, using ImmunoCAP (Phadia AB, Upsala, Sweden), were also performed. The results of allergologic tests are summarized in Table 1.

**Table 1:** Investigations performed in this patient (5 weeks after the anaphylactic episode)

Skin prick test^a^		Specific IgE^c^
Mite DP 30 × 15 mm	Cow’s milk: negative	Mite DP 16.00 kUA/L
Mite DF 25 × 10 mm	Egg yolk: negative	Mite DF 15.50 kUA/L
Mite *B. tropicalis* 24 × 12 mm	Egg white: negative	
	Shrimp: negative	Wheat 0.03 kUA/L
Contaminated Gogi® flour extract 1/5 w/v in saline 14 × 12 mm	Crab: negative	Omega-5 gliadin 0 kUA/L
	Clam: negative	
Newly opened Gogi® flour extract 1/5 w/v in saline: negative	Oyster: negative	Cow’s milk 0.02 kUA/L
	Soybean: negative	
	Peanut: negative	Egg white 0 kUA/L
Kapok 10 × 8 mm	Wheat grain: negative	Egg yolk 0 kUA/L
Cat 5 × 4 mm		
Dog 11 × 6 mm	Sticky rice flour extract 1/5 w/v in saline: negative	
Mouse epithelium 5 × 4 mm		
American cockroach 5 × 4 mm		
German cockroach 4 × 4 mm	Prick-to-prick test^b^	
	Coconut: negative	
Bermuda 5 × 4 mm		
Johnson 4 × 3 mm	Positive control: 8 × 8 mm	
Carelessweed 15 × 8 mm	Negative control: negative	
Acacia 5 × 4 mm		
Penicillium: negative		
Aspergillus: negative		
Alternaria: negative		

*B. tropicalis*, *Blomia tropicalis*; *DP*, *Dermatophagoides pteronyssinus*; *DF*, *Dermatophagoides farinae*; sIgE, specific immunoglobulin E; mm, millimeter; w/v, weight to volume ratio

^a^Normal saline and histamine (10 mg/mL) were used as negative and positive controls, respectively. We did not perform a latex skin test due to the unavailability of a standard solution

^b^Prick-to-prick test by using fresh fruit

^c^Solid-phase immunoassay: ImmunoCAP

## Discussion and conclusions

We report a case of dust mite ingestion-associated, exercise-induced anaphylaxis in a Thai patient. Most reported OMA cases developed symptoms immediately after eating mite-contaminated foods, but they can occur during physical exercise after the oral ingestion of the mites [6]. A recent review included 145 OMA cases from various regions [1]. However, dust mite ingestion-associated, exercise-induced anaphylaxis has only been reported twice [5, 6]. To the best of our knowledge, our report is the third reported case of dust mite ingestion-associated, exercise-induced anaphylaxis. We did not perform mite-contaminated food combined with exercise challenges due to the safety issue. However, 1 week before the anaphylactic event, our patient could tolerate the same bag of mite-infested flour without exercise at home.

An alternative explanation is house dust mite allergen level in the cooking flour could have increased with mite propagation [7]. However, the patient could tolerate 15 pieces of fried coconut rice cake without any reaction 1 week prior to the anaphylactic event compared with the 10 pieces associated with the event with exercise. Both the quantity of the food ingested and the 1-week interval of mite population increase should not have caused a significant increase in mite allergen ingestion associated with the anaphylactic event. This emphasizes the role of exercise as a cofactor to develop anaphylaxis in a patient who ingests mite-infested food. This reaction appears to be caused by heat-stable allergens, as cooking the flour does not seem to make a difference in terms of reactions in our case which is similar to the previous report [1].

OMA is associated with hypersensitivity to aspirin and NSAIDs (Non-steroidal anti-inflammatory drugs). A high prevalence of house dust mite allergic rhinitis and/or asthma has been observed in OMA patients [2, 8]. Although our patient had house dust mite allergic rhinitis, she had no NSAIDs hypersensitivity, which is similar to the two previously reported cases (Table 2). Whether NSAIDs could also be a cofactor for anaphylaxis development without exercise after ingesting mite-infested food similar to FDEIA (Food-dependent exercise-induced anaphylaxis) has not been well established [3].

**Table 2:** Case reports of dust mite ingestion-associated, exercise-induced anaphylaxis

	Our case	Adachi [6]	Sanchez-Borges [1]
Demographic data	29-year-old female	17-year-old male	16-year-old female
Country	Thailand	Japan	Spain
Comorbidities	Allergic rhinitis	None	Allergic rhinoconjunctivitis, asthma, atopic dermatitis, squid allergy
NSAIDs hypersensitivity status	None	None	None
Contaminated food	Fried coconut rice cake (Mixed cooking flour)	Pancake	Pancakes
Type of exertion	Jogging	Jogging	Playing soccer
Food intake-to-exercise interval (min)	60	90	30
Exercise-to-reaction interval (min)	20	Several	15
Symptoms	Breathlessness, angioedema, urticaria, rhinitis, chest tightness, hypotension	Abdominal cramp, breathlessness, angioedema, urticaria, oxygen desaturation	Breathlessness, angioedema
Mites	*Dermatophagoides farinae*	*Dermatophagoides farinae*	*Suidasia medanensis*
The method used to identify mites	Light microscopy	Light microscopy	Light microscopy
Allergic evaluation	Skin test positive to DP, DF, *B. tropicalis*, Contaminated cooking flour extract 1/5 w/v in saline Skin test negative to uncontaminated cooking flour extract 1/5 w/v in saline, wheat, oat, rye, barley, milk, egg, coconut (prick-prick)	sIgE positive to DP, DF, *G. destructor*, *T. putrescentiae*, *A. siro* sIgE negative to wheat, gluten, squid	Skin test positive to DP, DF, *B. tropicalis*, *A. siro*, *C. arcuatus*, *L. destructor*, *T. putrescientiae*, Contaminated pancake mix extract 1/5 w/v in saline Skin test negative to wheat, oat, rye, barley, milk, egg, Bermuda grass, ragweed

*A. siro*, *Acarus siro*; *B. tropicalis*, *Blomia tropicalis*; *C. arcuatus*, *Chortoglyphus arcuatus*; *DP*, *Dermatophagoides pteronyssinus*; *DF*, *Dermatophagoides farinae*; *L. destructor*, *Lepidoglyphus destructor*; *S. medanensis*, *Suidasia medanensis*; *T. putrescentiae*, *Tyrophagus putrescentiae*; NSAID, Non-steroidal anti-inflammatory drugs; sIgE, specific immunoglobulin E; w/v, weight to volume ratio

OMA is observed more frequently in geographical locations with high temperatures and relative humidity, favoring mite propagation. A series of OMA cases were reported from Venezuela, Spain, and Japan [1]. OMA is likely to be underreported in many countries with long periods of warm and humid weather, including Thailand. This condition is often overlooked and may be misdiagnosed as idiopathic anaphylaxis. The differential diagnoses include wheat allergy, allergy to hidden food allergens, food additives, and non-food allergens (e.g. drugs) [1]. In the case of suspicion of dust mite ingestion-associated, exercise-induced anaphylaxis, FDEIA should be excluded before making a diagnosis. OMA should be considered in mite allergic patients with food-induced allergic reaction who have no apparent allergy to the index food ingredients [6]. The diagnostic criteria for OMA were recently reviewed [1].

‘Gogi®’ is a well-known brand of cooking flours in Thailand which is composed of 90% wheat, 6% tapioca, 3% baking powder, and 1% of trace component. The previous report demonstrated that dust mite infestation of flour was dependent on the presence of wheat and a high ambient temperature in tropical regions [9]. It is recommended that cooking flours be kept in the refrigerator using sealed glass containers or plastic bottles. In tropical regions, it is recommended that cooking flour be kept refrigerated for no longer than 6 weeks to prevent significant mite propagation [1].

## Data Availability

Not applicable.

## Abbreviations

FDEIA: food-dependent exercise-induced anaphylaxis
IgE: immunoglobulin E
kAU/L: kilo allergy unit per liter
mmHg: millimetre of mercury
NSAIDs: non-steroidal anti-inflammatory drugs
OMA: oral mite anaphylaxis
